# Dynamic mRNA expression during chicken ovarian follicle development

**DOI:** 10.1093/g3journal/jkad237

**Published:** 2023-10-13

**Authors:** Hua Kui, Penghao Li, Tao Wang, Yingyu Luo, Chunyou Ning, Mengmeng Li, Siying Liu, Qing Zhu, Jing Li, Diyan Li

**Affiliations:** School of Pharmacy, Chengdu University, Chengdu 610106, People’s Republic of China; Jinxin Research Institute for Reproductive Medicine and Genetics, Chengdu Xi Nan Gynecological Hospital Co., Ltd., 66 Bisheng Road, Chengdu 610000, People’s Republic of China; Jinxin Research Institute for Reproductive Medicine and Genetics, Chengdu Xi Nan Gynecological Hospital Co., Ltd., 66 Bisheng Road, Chengdu 610000, People’s Republic of China; School of Pharmacy, Chengdu University, Chengdu 610106, People’s Republic of China; College of Animal Science and Technology, Sichuan Agricultural University, Chengdu 611130, Sichuan, People’s Republic of China; College of Animal Science and Technology, Sichuan Agricultural University, Chengdu 611130, Sichuan, People’s Republic of China; College of Animal Science and Technology, Sichuan Agricultural University, Chengdu 611130, Sichuan, People’s Republic of China; School of Pharmacy, Chengdu University, Chengdu 610106, People’s Republic of China; College of Animal Science and Technology, Sichuan Agricultural University, Chengdu 611130, Sichuan, People’s Republic of China; College of Agriculture, Kunming University, Kunming 650214, People’s Republic of China; School of Pharmacy, Chengdu University, Chengdu 610106, People’s Republic of China

**Keywords:** poultry, follicle development, granulosa cells, genetic, transcriptome, pathway

## Abstract

Ovarian follicle development is a complex and well-orchestrated biological process of great economic significance for poultry production. Specifically, understanding the molecular mechanisms underlying follicular development is essential for high-efficiency follicular development can benefit the entire industry. In addition, domestic egg-laying hens often spontaneously develop ovarian cancer, providing an opportunity to study the genetic, biochemical, and environmental risk factors associated with the development of this cancer. Here, we provide high-quality RNA sequencing data for chicken follicular granulosa cells across 10 developmental stages, which resulted in a total of 204.57 Gb of clean sequencing data (6.82 Gb on average per sample). We also performed gene expression, time-series, and functional enrichment analyses across the 10 developmental stages. Our study revealed that SWF (small while follicle), F1 (F1 hierarchical follicles), and POFs (postovulatory follicles) best represent the transcriptional changes associated with the prehierarchical, preovulatory, and postovulatory stages, respectively. We found that the preovulatory stage F1 showed the greatest divergence in gene expression from the POF stage. Our research lays a foundation for further elucidation of egg-laying performance of chicken and human ovarian disease.

## Introduction

The ovary is an endocrine and a terminally differentiated organ (Xiao et al. 2021) that plays a central role in the reproduction of vertebrates, and which encompasses a large number of follicles at different stages of development. The ovarian follicles are the basic unit of reproduction and are composed of an oocyte and surrounding granulosa cells (GCs). During ovarian follicle development, GCs provide the necessary nutrients and steroids to the oocytes (Sun et al. 2018). In light of the above, the study of chicken follicle development is of significant interest for several reasons, including: (1) Follicle development is a fundamental process in reproductive biology, whereby investigating the mechanisms underlying follicle development can provide valuable insights into the development and functioning of the reproductive system (Shen et al. 2020). Moreover, deciphering the molecular, cellular, and tissue-level processes implicated in follicle development enhances our comprehension of both normal reproductive ontogeny and pathological conditions (Franks et al. 2008); (2) research on follicle development contributes to the advancement of reproductive science by helping elucidate the intricacies of reproductive cycles, hormonal regulation, and molecular signaling pathways involved in follicle development. This knowledge may bring to light the fundamental principles of reproductive regulation and decisively contribute to advancements in assisted reproductive technologies, fertility treatments, and reproductive health management (De Vos et al. 2014; Xu et al. 2022); (3) abnormal folliculogenesis is associated with various reproductive disorders and infertility. Research on follicle development helps identifying the mechanisms underlying these conditions and improves the diagnosis of reproductive disorders, the development of therapeutic interventions, and reproductive health outcomes in animals and humans; (4) studying follicle development in animals is crucial for conservation efforts and breeding programs. This provides insights into the reproductive biology of endangered species and assists the development of suitable strategies for the conservation and management of domestic and wild populations; and (5) studies on follicle development across different animal species provide valuable insights into their evolutionary history and reproductive strategies, and helps identifying suitable animal models of human disease.

In mammals, the development of ovarian follicles is a highly selective process characterized by concurrent cellular events, including proliferation, differentiation, and apoptosis (Chen et al. 2005; Heng et al. 2020). Primordial follicles, consisting of a dormant oocyte surrounded by a single layer of flattened pre-granulosa cells, are formed during embryonic development, and remain quiescent until activated (Ting and Zelinski 2017). Once growth initiates, the activated primordial follicles with a single layer of flattened pre-granulosa cells surrounding the primordial oocytes develop into primary, secondary, and eventually antral follicles (Hsueh et al. 2015). Finally, a mature or antral follicle is formed, which is characterized by a fluid-filled cavity, called the antrum, within the granulosa cell layers. The oocyte reaches its maximum size and is surrounded by multiple layers of granulosa cells. This mature follicle is ready for ovulation, whereby it is released from the ovary to potentially be fertilized.

The embryonic ovaries of chickens develop symmetrically before sexual differentiation with no obvious left-right asymmetry (Ishimaru et al. 2008). During the embryonic stage, the left gonad increases significantly more than the right due to the presence of a higher number of germ cells encroaching the gonadal region (Mfoundou et al. 2021). With regard to ontogeny, the left gonad develops into a functional ovary while the right gonad regresses and degenerates (Jiang et al. 2022). Accordingly, chicken ovaries are unilaterally located on the left side of the body and produce sex hormones, including estrogen, progesterone, and small amounts of androgen (Matute and Kalkhoff 1973). The single follicle is the basic unit of the ovary (Lebbe and Woodruff 2013). In egg-layer hens, a unique follicular development process with large amount of yolk occurs in the cytoplasm of the oocyte (Zhou et al. 2020), and the ovarian follicles are usually arranged in a strict follicular hierarchy and can thus be divided into prehierarchical and hierarchical follicles (Deng et al. 2018). The former is also designated as small while follicle (SWF, <4 mm in diameter), large white follicle (LWF, 4–6 mm in diameter), small yellow follicle (SYF, 6–8 mm in diameter), and large yellow follicle (LYF, 9–12 mm in diameter). In contrast, the hierarchical follicles are sequentially classified based on their volume as F6 or F5, F4, F3, F2, and F1. Finally, there are 2–4 postovulatory follicles (POFs) after ovulation (Onagbesan et al. 2009).

Hormones are majorly responsible for regulating folliculogenesis and oogenesis. Follicular development is regulated by the hypothalamic–pituitary–gonadal axis, which involves the secretion of various hormones, such as gonadotropin-releasing hormone (GnRH), luteinizing hormone (LH), follicle-stimulating hormone (FSH), estrogen, progesterone, and prostaglandins. During the ovulation process, FSH and LH levels increase and promote follicular growth and maturation (Duan et al. 2023). During the development of the follicle, the surge in LH levels triggers ovulation and causes the follicle to rupture and the oocyte to be released into the oviduct. After ovulation, the remaining follicle becomes the corpus luteum, which produces progesterone. Progesterone levels increase during egg formation and are essential for the development of the shell gland (Pedernera et al. 1999). During folliculogenesis, the proliferation of GCs and hormone biosynthesis offer a crucial environment for follicular development.

Poultry production is one of the fastest growing industries worldwide, with a global production of over 135 million tons in 2021. The global population is projected to reach 8.5 billion by 2030, posing significant pressure on international poultry production (Xiong et al. 2018). In poultry breeds, high-efficiency follicular development implies huge economic output for the egg industry (Zhou et al. 2020). Previous studies demonstrated that poultry egg production is directly influenced by ovarian follicular development. For example, the parathyroid hormone-like hormone (PTHLH) is regulated by FSH in follicle selection, and has a positive effect on egg-laying traits in hens (Guo, Wang, et al. 2019). In fact, Guo, Liu et al. (2019) demonstrated that FSH promotes primordial follicle formation in chickens via the KIT signaling using in vivo FSH administration and in vitro ovarian tissue culture. Moreover, the laying hens that were fed on the naringin relive SWFs atresia that was induced by oxidative stress and maintain egg-laying performance of aging low-yielding hens by reducing oxidative stress (Bao et al. 2022). Dietary supplementation of N-carbamylglutamate (NCG) at a 1% concentration for 14 days promotes ovarian follicle development in chickens by enhancing angiogenesis, as evidenced by increased follicular size (Ma et al. 2020). Other studies also suggested that some genes affect follicular development, including GDF9 with an essential role in folliculogenesis and granulosa cell proliferation, which leads to improved follicle development and fertility (Hlokoe et al. 2022).

Egg-laying hens are considered an ideal model organism for ovarian cancer (OC) (Johnson and Giles 2013), a lethal malignancy affecting women that includes the highest case-to-death ratio among gynecological cancers (Barua and Bahr 2022). Understanding the molecular mechanisms underlying follicular development can also provide insights into the pathogenesis of reproductive disorders in poultry, but also in humans. In fact, the domestic laying hen is the only nonhuman animal with a high prevalence of spontaneous OC, which provides an opportunity to study the genetic, biochemical, and environmental risk factors involved in tumor initiation, progression, and histological origin. In comparison to traditional animal models (e.g. rodents), laying hens develop OC spontaneously, with remarkable similarities to OC in women regarding tumor histology, OC dissemination, immune responses, and risk factors (Barua and Bahr 2022). Despite anatomical and physiological differences between species, the etiology and pathogenesis of ovarian cancer are similar between chicken and humans. Previous reports showed that ovarian cancer in hens is highly malignant and follows a pattern of dissemination, metastasis, and formation of ascites similar to those observed in human patients (Tudrej et al. 2019). In addition, domestic chicken (*Gallus gallus domesticus*) has a smaller genome, shorter life cycle, and lower cost, making it an ideal candidate for OC drug testing. Hakim et al. (2009) used a series of experiments that revealed ovarian adenocarcinomas in laying hens and women share similar changes in the p53 tumor suppressor gene, as well as in the ras and HER-2/neu oncogenes. In addition, Lim et al. (2012) investigated the functional role played by the SERPINB3 (serine protease inhibitors) gene in human epithelial ovarian cancer using chicken as an animal model. The authors found chicken and human ovarian cancer cell lines have patterns of *SERPINB3* expression. Another study showed that chicken ovarian tumors and cultured ovarian cancer cells express CA125, which is a hallmark of ovarian cancer in women (Jackson et al. 2007).

Deep transcriptome sequencing (RNA-seq) (Mortazavi et al. 2008; Wang et al. 2009) using next-generation sequencing (NGS) technologies provides unprecedented opportunities for studying the molecular basis of folliculogenesis. Previously, Shen *et al*. used RNA sequencing to profile circRNAs and mRNAs in theca cells from small yellow follicles (SYF), the smallest hierarchical follicles (F6), and the largest hierarchical follicles (F1). The findings revealed an enrichment in both differentially expressed circRNAs and mRNAs in pathways associated with reproduction—including the TGF-β signaling pathway, oocyte meiosis, and vascular smooth muscle contraction (Wang, Zhao, et al. 2017). Moreover, Wang, Chen et al. (2017) used RNA sequencing in small yellow follicles (SYF) and demonstrated that Wnt4 plays an important role in chicken follicle selection by stimulating GCs proliferation and steroidogenesis. Finally, Fan et al. (2019) used single-cell RNA sequencing to identify distinct types of granulosa and theca cells in the human ovary.

Beyond the aforementioned evidence, previous studies also highlighted the critical role of nutrient (e.g. protein and amino acids) supplementation (Jiang et al. 2019) and hormones on improving egg production in laying hens. However, these studies focused solely on either 1 or a few phases (Wang, Chen, et al. 2017), or instead a handful of genes (Wang, Zhao, et al. 2017). Hence, a truly holistic perspective on ovarian follicle development in chicken is currently missing. In this study, we employed RNA-seq to study the transcriptome profiles of chicken ovarian follicles at 10 developmental stages, including prehierarchical follicles (SWF, LWF, SYF, and LYF), preovulatory follicles (F5, F4, F3, F2, and F1), and postovulatory follicles (POFs). These datasets provide valuable information for our understanding of follicle development in laying hens, and identifies potential target genes for the breeding of highly productive egg-layer hens.

## Materials and methods

### Ethics statement

All animal protocols were approved by the Institutional Animal Care and Use Committee of Sichuan Agricultural University (protocol number B20171910). The methods were performed in accordance with the approved guidelines.

### Animals and sample collection

We used healthy Luhua hens at the peak laying period (31 weeks of age) from the Experimental Chicken Farm of Sichuan Agricultural University. The animals were euthanized by intravenous injection of 2% pentobarbital sodium (25 mg/kg of body weight). The ovarian follicles were categorized based on the size of the follicle diameter. The follicles of each developmental stage were then counted and their diameter measured using a vernier caliper.

Follicular granulosa cell layers of the whole reproductive cycle, including prehierarchical follicles (SWF, LWF, SYF, and LYF), preovulatory follicles (F5, F4, F3, F2, and F1), and postovulatory follicles (POFs), were collected according to the methodology described by Gilbert et al. (1977). All separated samples were promptly frozen in liquid nitrogen and stored at −80°C until used for RNA-seq library preparation.

### Total RNA isolation, library preparation, and sequencing

Total RNA was extracted from each sample using the RNAiso Plus reagent (TaKaRa, Otsu, Shiga, Japan) following manufacturer's instructions. We estimated the integrity and quality of the total RNA using a Bioanalyzer 2100 system (Agilent Technologies, Palo Alto, CA, USA) with an RNA 6000 Nano kit. Subsequently, we generated 30 strand-specific libraries (total of 10 stages with 3 biological replicates in each stage) after depleting rRNA using the Ribo-Zero Gold Kit (Illumina, San Diego, CA, USA). The libraries were sequenced on an Illumina HiSeq X Ten platform (Illumina Inc., San Diego, CA, USA) using paired-end 150 bp sequencing reads (PE150) at the Novogene Corporation (Beijing, China).

### Gene expression levels and time-series analysis

High-quality reads were obtained by removing low-quality reads, adapter sequences, null reads, and ribosomal (r)RNA reads from the raw data. The processed reads were mapped to the chicken reference genome (GRCg6a) using HISAT2 2.1.0 (Kim et al. 2015). The mapped reads were assembled and then quantified for gene expression based on the FPKM values of each mRNA using StringTie v1.3.3 (Pertea et al. 2015). The mRNAs that exhibited FPKM > 0.5 in at least 2 biological replicates were filtered for expressed protein-coding genes, and log_2_-transformed values of FPKM + 1 were used for further analysis. Spearman correlations were calculated across developmental stages. The differentially expressed genes (DEGs) were identified using DEseq2 (Love et al. 2014) based on the read count data. The significant DEGs were screened with a false discovery rate < 0.05 and |fold change| > 1.5 as cutoffs. MaSigPro (v 3.12) (Conesa et al. 2006) was used to identify the genes with dynamic temporal expression profiles.

### GO & KEGG pathway enrichment analysis

Gene Ontology (GO) terms and Kyoto Encyclopedia of Genes and Genomes (KEGG) pathway analyses were performed with Metascape (http://metascape.org/gp/index.html#/main/step1) (Zhou et al. 2019). The GO terms with a *P* < 0.01 containing ≥ 3 genes were considered as statically significant.

### Statistical analyses

All statistical analyses were performed using a 2-tailed Student's t-test or a Mann–Whitney U test in GraphPad Prism 8 or R.

## Results

### Follicle diameters at all levels and read alignments

Table 1 displays the follicle numbers and diameters of the prehierarchical, preovulatory, and postovulatory follicles using in this study.

**Table 1:** . Statistics of follicle diameter at all levels.

Follicles	Numbers	Diameter (mm)
SWF	>15	<4
LWF	7–12	4.28 ± 0.24
SYF	4–6	8.50 ± 0.87
LYF	1–3	12.77 ± 1.38
F5	1	20.74 ± 1.39
F4	1	26.33 ± 0.98
F3	1	29.20 ± 0.34
F2	1	32.22 ± 1.09
F1	1	37.16 ± 1.18

**Fig. 1.**
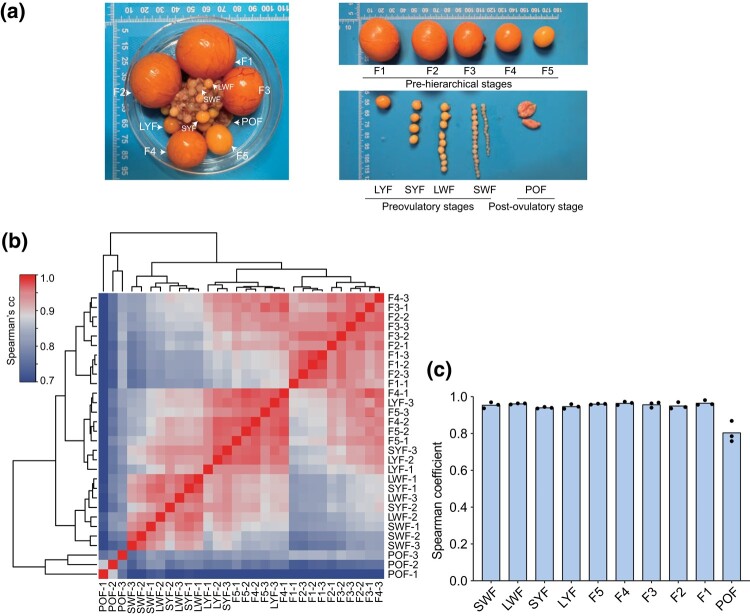
The structure of ovary and expression profiles of mRNAs during chicken follicle development. a) The structure of the ovary at the peak period of egg production in Luhua chicken samples. b) Heatmap of Spearman's correlation coefficient between pairs of samples. c) Histogram of Spearman's correlation coefficients within each sample.

To systematically explore the transcription patterns during chicken follicle development, we used RNA-seq to profile the mRNAs of follicular GCs at 10 distinct stages, including prehierarchical (SWF, LWF, SYF, and LYF), preovulatory (F5, F4, F3, F2, and F1), and postovulatory follicles (POFs) (Fig. 1a). A total of 219.34 Gb of sequencing data was generated from 30 raw datasets representing 3 biological replicates for each stage. The average sequencing data in each sample were 7.31 Gb. After a series of quality control steps, including data preprocessing, the removal of low-quality sequences, adapters, and rRNA sequences, we obtained 1,389.26 million high-quality reads (clean reads), corresponding to a total of 204.57 Gb of clean data (6.82 Gb on average per sample), approximately 93.27% of the original data. The average Q30 (proportion of base calling accuracy > 99.9%) of all samples was approximately 92.45% (Supplementary Table 1), indicating suitable quality of the generated RNA-seq data for subsequent bioinformatic analysis. After filtering out genes with low expression levels, we identified 15,197 genes that were substantially expressed in at least 2 replicates (FPKM > 0.5). A heatmap of the correlation showed high reproducibility within biological replicates (Spearman *R* > 0.80) (Fig. 1b and c).

### Differential gene expression analysis in ovarian follicle development

**Fig. 2.**
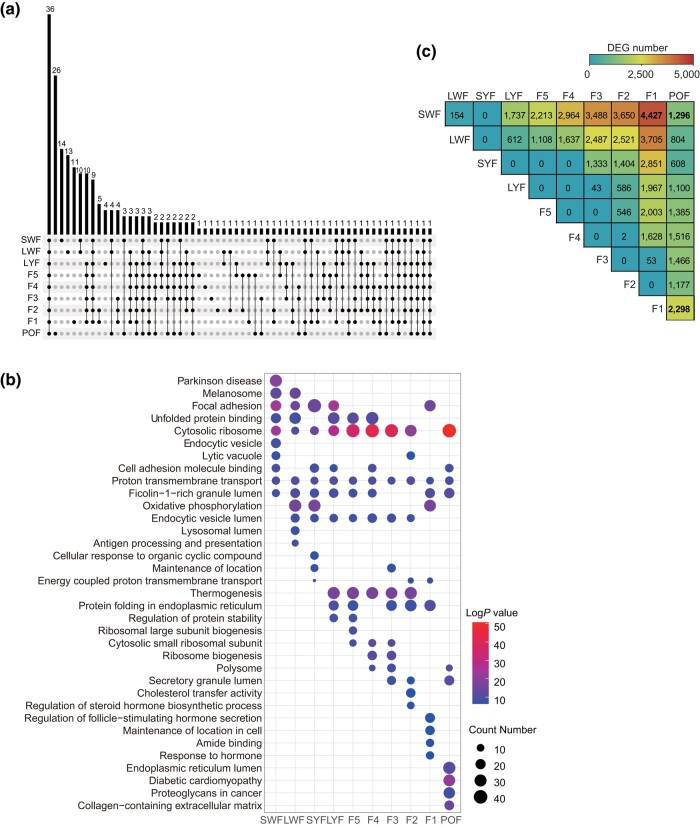
Differential gene expression during chicken follicle development. a) UpSet plot of the top 100 most highly expressed genes at each stage. The number of co-expressed genes in each set appears above the column, while the stages shared are indicated in the graphic below the column. b) The top 10 significantly enriched Gene Ontology-Biological Process (GO-BP) terms of the top 100 most highly expressed genes at each stage. c) Heatmap of DEGs in pairwise comparisons between stages. DEGs were identified with a threshold of |log_2_FC| > 1.5 and a false discovery rate (FDR) < 0.05.

To distinguish the transcriptomic divergence at different stages, we analyzed the top 100 highly expressed genes (Supplementary Table 2) and identified 36 (involve 2 novel genes) that were co-expressed across all stages (Fig. 2a, Supplementary Table 3). Of these, we identified 16 mitochondrial genes, including *ND1*, *ND2*, *ND3*, *ND4*, *ND5*, *ND6*, *ND4L*, *CYTB*, *COX1*, *COX2*, *COX3*, *ATP6*, and *ATP8*; 18 autosomal genes, including *SPARC*, *GAPDH, ACTB*, *GNS*, *EEF1A*, *FTH1*, *ZP3*, *CALR*, *HSP90AA1*, *PPIB*, *RPS3A*, *EIF4A2*, *RPLP1*, *CTSB*, *APLP2*, *RPS10*, *RPL23*, and *RPL6*. These genes are involved in positive regulation of cell adhesion and the regulation of translation.

We also found their stage-specific genes in each stage of follicle development, mostly in the case of SWF, F1, and POF (Fig. 2a). The functional enrichment analysis showed that most of these co-expressed genes are involved in metabolic and cell adhesion processes, such as cytoplasmic translation (GO:0002181), adherens junction (GO:0005912), and peptide biosynthetic process (GO:0043043) (Fig. 2b). The stage-specific genes are associated with reproductive, signaling, and localization processes (Fig. 2b), which reflect distinct biological functions in chicken follicular GCs during development.

For comparison, we also performed pairwise differential expression analysis for the 10 developmental stages, but found no differences in gene expression between contiguous stages of development (Fig. 2c). Comparing the DEGs between prehierarchical follicle and postovulatory follicle, we found that SWF contains the most DEGs (1,296) when compared to the POF stage. Comparing the DEGs between preovulatory follicle and postovulatory follicle, the stage of F1 showed the greatest divergence (2,298) in gene expression also when compared to the POF stage. The highest number of DEGs (4,427) was noticed between the stage of SWF and F1. Accordingly, the SWF, F1, and POF stages were considered as best representing the transcriptional changes associated with prehierarchical, preovulatory, and postovulatory stages, respectively.

### Differential gene expression analysis

**Fig. 3.**
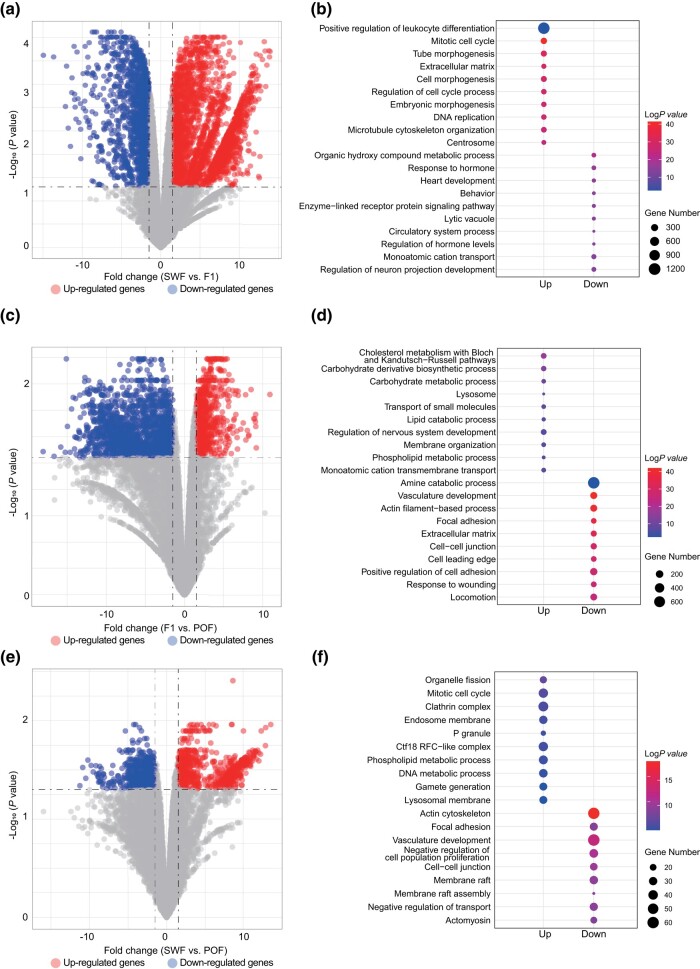
Differential gene expression analysis in SWF, F1, and POF. Volcano plots showing significantly differentially expressed genes (DEGs) (|Fold change|>1.5, adjusted *P*-value < 0.05) in a) SWF vs F1, b) F1 vs POF, and c) SWF vs POF. The top 10 significantly enriched Gene Ontology-Biological Process (GO-BP) terms of the DEGs in d) SWF vs F1, e) F1 vs POF, and f) SWF vs POF.

As previously described, the SWF, F1, and POF stages were considered as best representing the transcriptional changes associated with prehierarchical, preovulatory, and postovulatory stages, respectively (Fig. 2c, Supplementary Table 4). Genes up-regulated or down-regulated were further analyzed in the 3 representative stages. There were 2,922 up-regulated genes and 1,505 down-regulated genes detected for SWF vs F1 (Fig. 3a). The up-regulated genes were enriched in the pathway of “mitotic cell cycle” (GO:0000278), and the down-regulated genes were enriched in the pathway of “regulation of hormone levels” (GO:0010817) (Fig. 3b, Supplementary Table 5). The genes enriched in “mitotic cell cycle” pathway, *BUB1B* and *CDC20*, were involved in cell cycle regulation (Nakata et al. 2017). There were 759 up-regulated genes and 1,539 down-regulated genes detected for F1 vs POF (Fig. 3c). The down-regulated genes were enriched in the pathway of “vasculature development” (GO:0001944) (Fig. 3d, Supplementary Table 5). There were 821 up-regulated genes and 475 down-regulated genes detected for SWF vs POF (Fig. 3e). The functional enrichment analysis showed that up-regulated genes were enriched in “mitotic cell cycle” (GO:0048285), and the down-regulated genes were enriched in “vasculature development” (GO:0001944) (Fig. 3f, Supplementary Table 5). Interestingly, in the stage of POF, the “vasculature development” pathway is down-regulated (Fig. 3d and e).

**Fig. 4.**
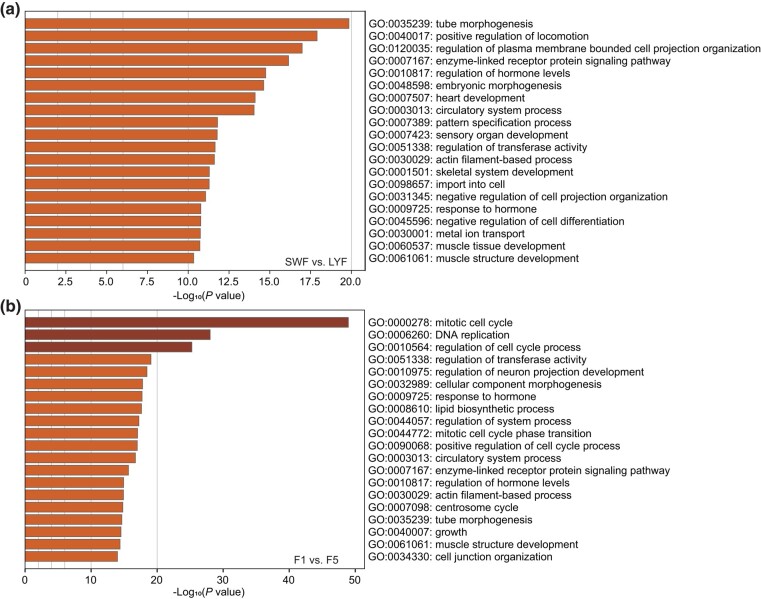
The top 20 significantly enriched Gene Ontology-Biological Process (GO-BP) terms of the DEGs in a) SWF vs LYF and b) F1 vs F5.

Meanwhile, we also focused on the DEGs within prehierarchical stage (SWF vs LYF) and preovulatory stage (F1 vs F5). There were 1,737 and 2,003 DEGs individual. The DEGs of SWF vs LYF were enriched in the pathway of “enzyme-linked receptor protein signaling pathway” (GO:0007167), “regulation of hormone levels” (GO:0010817), and “actin filament-based process” (GO:0030029) (Fig. 4a, Supplementary Table 5). The DEGs of F1 vs F5 were enriched in the pathway of “mitotic cell cycle” (GO:0000278), “response to hormone” (GO:0009725), and “actin filament-based process” (GO:0030029) (Fig. 4b, Supplementary Table 5). Most of these pathways were closely related to folliculogenesis (Bell and Dutta 2002; Gosden and Lee 2010).

### Temporal expression patterns of the chicken follicle transcriptome during development

**Fig. 5.**
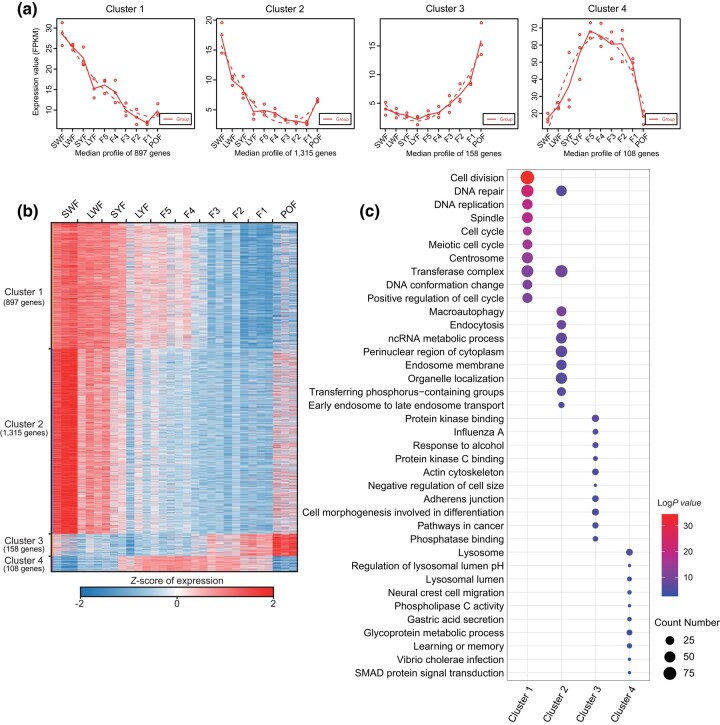
Transcriptomic dynamics during chicken follicle development. a) Clustering of mRNAs using maSigPro based on the median mRNA expression levels. b) Heatmap of the normalized mRNA expression levels (Z-score) in each cluster from a). c) Gene Ontology (GO) analysis of mRNAs for each cluster.

The spatiotemporal dynamics of gene expression reflect changes in cell function during development. Here, we used maSigPro30 to cluster all genes based on the time course of follicle development. Accordingly, a total of 2,478 genes were grouped into 4 clusters that significantly changed during follicle development (Fig. 5a; Supplementary Table 6). The 4 clusters highlight different temporal patterns of gene expression during chicken follicle development. Specifically, we identified 897 genes in cluster 1, which showed decreased expression levels over the course of development (Fig. 5a and b); 1,315 genes in cluster 2, which showed the highest expression levels in the SWF. The expression levels of these genes decreased until follicle selection, and continued to be low at the hierarchical stage, finally increasing in POF; 158 genes in cluster 3, which increased their expression levels over the course of development, especially in the late preovulatory and postovulatory stages; and 108 genes in cluster 4, which exhibited highly stable expression at the preovulatory stage and relatively low expression levels at the prehierarchical and postovulatory stages (Fig. 5a and b). GO analysis revealed obvious functional enrichment differences between clusters (Fig. 5c). Genes in cluster 1 were mostly involved in the cell cycle, and chromosomal region, and localization. A considerable number of genes in clusters 2 and 3 were associated to autophagy and apoptosis, corresponding to the major stage of ovarian follicle degeneration caused by the apoptosis of GCs. Finally, genes in cluster 4 were mostly involved in growth and developmental processes, indicating that the proliferation and functional activity of GCs are accompanied by the maturation of hierarchical follicles (Fig. 5c). These substantial transcriptomic dynamics demonstrate distinct clustering of gene expression during chicken follicle development.

## Discussion

The pursuit of high egg production has always been the primary goal for poultry breeders. This important trait depends on efficient ovarian development and ovulation, whereby optimal reproductive efficiency in domestic hens can be achieved through enhanced follicular development. Typically, preovulatory follicles are selected from a pool of growing follicles, which are classified based on size (3–5 mm or 6–8 mm) or color (large white or small yellow follicles). Follicle-stimulating hormone (*FSH*) facilitates the selection of follicles, and previous research showed that treatment with pregnant mare serum gonadotropin (*PMSG*) or *FSH* increases the number of developing follicles. The granulosa layer of developing follicles expresses follicle-stimulating hormone receptors (*FSHR*), and the abundance of mRNA for these receptors changes as follicles mature. Previous studies revealed that the mRNA for *FSHR* is highest in the granulosa layer of 6–8 and 9–12 mm follicles (You et al. 1996), with 1 follicle in the pool of 6–8 mm follicles containing significantly more mRNA for *FSHR* than the other follicles in this size category. This observation suggests that this follicle may be selected next to move into the larger group (Woods and Johnson 2005). As follicles progress through the hierarchy, the granulosa layer becomes capable of producing an increasing amount of progesterone, which eventually results in a surge of luteinizing hormone and the start of ovulation (Robinson and Etches 1986). Follicular development is thus an important process that affects a high number of egg production traits in the poultry industry. Hence, the molecular mechanisms associated with follicular development in chickens have gained significant attention in poultry reproduction biology (Li et al. 2022). Obtaining insights into this sophisticated process of gene transcription and expression during follicle development can enhance our understanding on the reproductive physiology of hens and improve laying performance, also providing benefits for medical research on ovarian cancer using these animal models (Fu et al. 2014). Here, we performed RNA-seq to systematically investigate the gene expression profiles of granulosa cells in ovarian follicles across the whole reproductive cycle. Substantial transcriptomic dynamics showed distinct gene expression patterns corresponding to specific stages of ovarian follicle development.

In this study, we provide high-quality RNA sequencing data with a total of 204.57 Gb of clean data, and an average of 6.82 Gb of per sample. The genome size of chicken (*Gallus gallus domesticus*) is 1.1 Gb (GRCg6a), significantly smaller than the genome size of pig (*Sus scrofa*, susScr11, 2.5 Gb) and human (*Homo sapiens*, GRCh38, 3.08 Gb). Smaller genome size means higher sequencing depth in a certain amount of data. Higher sequencing depths mean higher cost and higher detection sensitivity and reliability.

The spearman correlation analyses showed high reproducibility (Spearman *R*>0.96) within biological replicates of other stages, except for the POF stage (Spearman *R* = 0.86) (Fig. 1c). A possible explanation for this observation is the insufficient number of biological replicates for this stage. After ovulation, the granulosa cells remaining in the follicle undergo luteinization (Johnson 2012) and apoptosis (Fan et al. 2008). Apoptosis triggers dramatic changes in cellular physiology including dramatic inhibition of transcription, RNA decay, and mRNA translation (Lee et al. 2021). Here, we found that the SWF, F1, and POF stages contain the largest transcriptome differences of any stage of follicle development (Fig. 2a, Fig. 4). These observations are consistent with those of previous reports (Shen et al. 2019), and reflect the corresponding functional characteristics of follicle development at different physiological stages.

We also focused on the up- and down-regulate genes in DEGs of SWF vs POF, SWF vs F1, and F1 vs POF. During the SWF stage, follicles are classified as primary, with the granulosa cells arranging themselves into a monolayer structure. During follicle development, granulosa cells undergo a transition from single-layered to multilayered. From the secondary LWF and SYF follicle stages to the selection phase follicles accumulate yolk material, increase in size, and granulosa cells transition from a multilayered to a single-layered structure. GCs ultimately reach the mature follicle stage during F1 and start ovulation. SWF and F1 represent critical follicle development stages characterized by significant morphological and functional changes in granulosa cells, resulting in the highest number of stage-specific gene expression, as observed in our transcriptomic findings (Fig. 2). Hence, compared to other stages, the functional changes in granulosa cells occurring during the POF stage are ultimately reflected in the specificity of their gene expression. Accordingly, the top 100 DEGs at the POF stage are mainly associated with apoptotic or autophagy-related signaling pathways, such as NIK/NF-κB signaling regulation, regulation of cytokine production involved in immune response, and PI3K-Akt signaling pathway. DEGs down-regulated in SWF vs POF and F1 vs POF both enriched in the pathway of “vasculature development”. This is in accordance with the atrophic follicles after ovulatory.

Analysis of mRNA temporal expression patterns (Fig. 5a) showed that cluster 2 genes exhibited primarily high expression levels in prehierarchical follicles, followed by a gradual decrease in preovulatory follicles. However, the expression of these genes significantly increased during the POF stage. In addition to pathways associated with apoptosis or autophagy, our functional enrichment analysis also revealed pathways related to hormone synthesis and secretion, such as steroid hormone secretion, as well as biological processes, including steroid synthesis. In mammals, the synthesis of steroids is primarily carried out by the corpus luteum or the placenta. In poultry, however, this process is mainly performed by granulosa cells located in the preovulatory follicular tissue (Huang et al. 1979). Following ovulation, poultry granulosa cells do not undergo immediate apoptosis. Instead, GCs remain attached to the follicular membrane and survive for several days. Previous research indicates that postovulatory granulosa cells play a crucial role in ensuring the smooth progression of the subsequent egg-laying cycle in poultry (Gilbert et al. 1978). Moreover, it is widely accepted that granulosa cells continue to secrete crucial hormones and cell factors even after ovulation, including estrogen and prostaglandins that act on preovulatory follicles (e.g. SWF) and stimulate their development (Lin et al. 2018). In cluster 4, the temporal expression pattern of genes rapidly increases from SWF to prior the selection of follicles, reaching its peak during the hierarchical stage. However, after ovulation, there is a sharp decline in the expression of genes in cluster 4, which are primarily associated with calcium ion transport, transmembrane export, regulation of cell growth, glycoprotein biosynthetic process, and epithelial cell proliferation.

## Conclusions

Our study revealed the dynamic transcriptome expression patterns during the development of chicken ovarian follicles by RNA-seq. These results provide a theoretical basis for improving the productivity of laying hens and developing models of human ovarian cancer. In the future, the development of single-cell RNA sequencing technologies will help clarifying cell type composition and function at each stage of ovarian follicle development, providing new perspectives on folliculogenesis.

## Supplementary Material

jkad237_Supplementary_Data

## Data Availability

The RNA-seq data of chicken ovarian follicle generated in this study have been deposited in the Gene Expression Omnibus (GEO) database under accession code “GSE229714”. Supplemental material available at G3 online.

## Code availability

The code for producing the results explained in this research is available at https://github.com/HuaKui17/Git-Repository/tree/main.

Supplementary Table 1: data summary of RNA-seq for all 30 samples; Supplementary Table 2: top 100 expressed genes at each stage; Supplementary Table 3: the co-expression genes in top 100 most expressed genes; Supplementary Table 4: list of differentially expressed genes; Supplementary Table 5: the enriched Gene Ontology-Biological Process (GO-BP) terms of the DEGs; Supplementary Table 6: list of genes for each cluster.
